# Limited sampling hampers “big data” estimation of species richness in a tropical biodiversity hotspot

**DOI:** 10.1002/ece3.1405

**Published:** 2015-01-21

**Authors:** Kristine Engemann, Brian J Enquist, Brody Sandel, Brad Boyle, Peter M Jørgensen, Naia Morueta-Holme, Robert K Peet, Cyrille Violle, Jens-Christian Svenning

**Affiliations:** 1 Ecoinformatics & Biodiversity, Department of Bioscience, Aarhus UniversityNy Munkegade 114, Aarhus C, DK-8000, Denmark; 2 Ecology & Evolutionary Biology, University of ArizonaBiosciences West 310, Tuscon, Arizona, 85721; 3 Missouri Botanical GardenP.O. 299, St. Louis, Missouri, 63166-0299; 4 Integrative Biology, University of California3040 VLSB, Berkeley, California, 94720-3140; 5 Department of Biology, University of North CarolinaChapel Hill, North Carolina, 27599-3280; 6 CEFE UMR 5175, CNRS – Université de Montpellier – Université Paul-Valéry MontpellierEPHE 1919 route de Mende, Montpellier, CEDEX 5, F-34293, France

**Keywords:** Ecuador, rarefaction, resampling, richness estimation, sampling effort

## Abstract

Macro-scale species richness studies often use museum specimens as their main source of information. However, such datasets are often strongly biased due to variation in sampling effort in space and time. These biases may strongly affect diversity estimates and may, thereby, obstruct solid inference on the underlying diversity drivers, as well as mislead conservation prioritization. In recent years, this has resulted in an increased focus on developing methods to correct for sampling bias. In this study, we use sample-size-correcting methods to examine patterns of tropical plant diversity in Ecuador, one of the most species-rich and climatically heterogeneous biodiversity hotspots. Species richness estimates were calculated based on 205,735 georeferenced specimens of 15,788 species using the Margalef diversity index, the Chao estimator, the second-order Jackknife and Bootstrapping resampling methods, and Hill numbers and rarefaction. Species richness was heavily correlated with sampling effort, and only rarefaction was able to remove this effect, and we recommend this method for estimation of species richness with “big data” collections.

## Introduction

Growing concern about the status and future of the world’s biodiversity in the face of human-induced climate and land-use change has focussed attention on the need to mitigate these negative effects (Botkin et al. 2007). At the same time, limited funds have raised demands for resource-efficient conservation tactics (Margules and Pressey 2000). A primary goal of large-scale conservation efforts is to conserve as much biodiversity as possible with minimum investment (Myers et al. 2000). This requires comparable and reliable estimates of species richness across large geographic scales (Ibáñez et al. 2006). However, species distributions are often poorly understood (Wallacean short-fall) and many species remain undescribed (Linnaean shortfall) (Whittaker et al. 2005; Sheth et al. 2012; Ter Steege et al. 2013). This is particularly true with respect to the tropics (Ferrier 2002). Sampling methods and sampling intensity have been inconsistent across space and time, making the calculation of accurate and comparable species richness estimates problematic (Colwell et al. 2012).

Museum specimens are an important source of information for studies of biodiversity (Shaffer et al. 1998). In recent years, many museums have undertaken digitization of their collections and have been making these data publically available through internet sources such as the Global Biodiversity Information Facility (GBIF, http://www.gbif.org/). Since the 1990s, the number of published studies using “big data” from collections of herbarium specimens to investigate biogeographic patterns or environmental changes has increased almost exponentially (Lavoie 2013). However, these records are the result of years of different researchers working with different aims and methodologies and as a result may suffer from various types of sampling bias. Site accessibility is highly correlated with the number of specimens in a given area, and most specimens are found in close proximity to roads, cities, and rivers (Reddy and Da 2003; Kadmon et al. 2004). Sampling may tend to be higher for certain functional or taxonomic groups that have received special attention, and this bias may also potentially vary spatially. Such biases may cause joint spatial and taxonomic biases that must be considered to accurately estimate species richness from museum specimens (Mateo et al. 2013).

Development of new methods to correct for sampling bias in the estimation of spatial and temporal variation in species richness has received much focus in recent years (Ibáñez et al. 2006; Colwell et al. 2012). The simplest measure of biodiversity is the number of species observed within a geographic unit, but this estimate is strongly affected by sample size (number of specimens) (Hellmann and Fowler 1999). The importance of sampling bias is also related to spatial scale. At coarser resolution, data coverage is improved and sampling biases should be weaker. However, coarse-grained studies are not always useful for directing conservation efforts (Ferrier 2002), and in the assessment of drivers of species richness, the choice of scale can change the estimated importance of individual factors (McGill 2010).

The biotas of tropical areas are generally much less studied than those of temperate regions (Ferrier 2002). However, it is clear that certain tropical regions contain the highest plant species density on Earth. For this reason, tropical areas have often been the focus of studies aimed at optimizing global conservation efforts (Myers et al. 2000; Malcolm et al. 2006). Ecuador has been identified as a tropical biodiversity hotspot (Jørgensen and León-Yánez 1999; Conservation International 2007) and has been shown to be particularly well sampled compared to other tropical New World countries (Schulman et al. 2007; Ulloa et al. 2011). Ecuador also has very heterogeneous environmental conditions, making it an ideal region for assessing the effects of environmental gradients on patterns of tropical species richness (Skov and Borchsenius 1997; Distler et al. 2009; Jiménez et al. 2009) relative to the effect of sampling.

The aims of this study are threefold: (1) document broad-scale spatial patterns of species richness for a tropical biodiversity hotspot, (2) determine the effect of geographic scale and sampling bias on estimates of species richness and their relationships with environmental factors, and (3) evaluate the effectiveness of different methods for correcting sampling bias. We hypothesize that (1) at higher resolution (smaller grid cell size), estimates become more biased due to decreased and inconsistent numbers of specimens resulting in poorer model fit and imprecise parameter estimates, (2) methods of greater mathematical complexity can result in improved species richness estimates, and (3) the effects of predictor variables on species richness are scale dependent.

## Materials and Methods

### Predictor variables

We chose topographic heterogeneity, annual mean temperature, and mean yearly precipitation as potential environmental drivers of species richness patterns, as these have been identified by previous authors as important drivers, both generally (Kreft and Jetz 2007) and in Ecuador (Skov and Borchsenius 1997). Elevation data were downloaded from the CGIAR Web site (20 September 2008, http://srtm.csi.cgiar.org/) at 90 × 90 m resolution and used to calculate topographic heterogeneity defined as range of elevation. Mean annual temperature and annual precipitation data were downloaded from the WorldClim Web site (12 November 2011, http://www.worldclim.org/bioclim) at 1 × 1 km resolution (Hijmans et al. 2005).

### Specimen and sample data

Georeferenced plant species specimens for Ecuador were downloaded from the Botanical Information and Ecology Network (BIEN) (Enquist et al. 2009; http://bien.nceas.ucsb.edu/bien/). The data contain 205,735 specimens from Ecuador of 15,788 species. All species names in the BIEN database are taxonomically standardized and synonyms updated to currently accepted names with the Taxonomic Name Resolution Service (version 1; Boyle et al. 2013), with Tropicos® as the taxonomic authority (http://www.tropicos.org). Furthermore, all georeferenced specimens in the BIEN database are geoscrubbed to ensure high reliability of the coordinates.

Sampling effort was measured as the number of specimens per sample (here defined as a grid cell) and compared with the environmental variables effect on species richness estimates. We assume that the relationship between the true species richness and sampling effort is weak and if an estimation method is successful in removing sampling bias, expectation is to see weaker correlations between species richness and sampling effort than between species richness and the environmental variables. To further test the influence of sampling effort, we reran the models on subsets of the data by excluding cells with fewer than 20, 50, or 100 samples. However, this did not significantly change the results of the regression analyses and these results are only shown in the supplementary material (Table S1 in Supporting Information).

**Figure 1:**
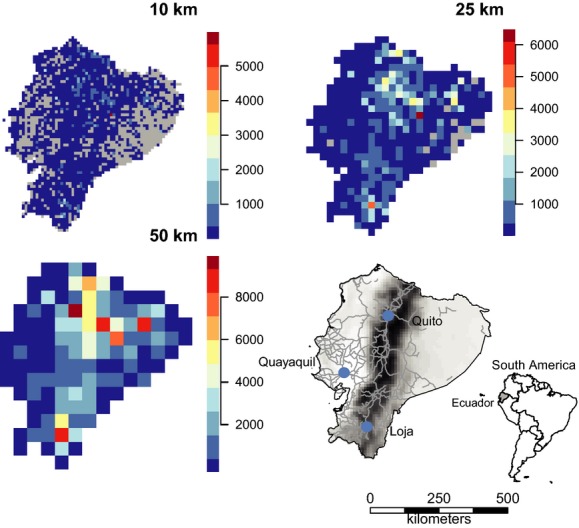
Sampling intensity across Ecuador at three different scales. Sampling intensity was calculated as the number of point observations within a grid cell. Gray cells lack any observations. Species observations were projected to the Lambert Azimuthal equal-area projection before being rasterized to avoid any effect of area on species richness estimates. Also shown is Ecuador with major roads and the cities with major herbariums. The road and cities layer was downloaded from the Global Administrative Areas database (21 November 2013, http://www.gadm.org).

To test the effect of grid size on relationships between species richness and environmental drivers, species and environmental data were rasterized and analyzed at different grid cell sizes: 10 × 10, 25 × 25, and 50 × 50 km, respectively. In the following, species richness at these three scales is considered as comparisons of gamma diversity. We discuss species richness patterns for Ecuador in three major regions for comparison with other studies: the central Andean region going through the middle of Ecuador, the western coastal region, and the eastern Amazonian region (Fig. 1). The characteristics of the regions are thoroughly described by Jørgensen and León-Yánez (1999). The georeferenced specimens were projected to the Lambert Azimuthal equal-area projection to ensure equal grid cell area, thereby avoiding area effects on species richness estimates. Topographic heterogeneity and temperature were highly correlated (Pearson’s *r* = −0.65 at 10 × 10 km scale) and were separated into two individual multiple regression model sets, each combined with precipitation and sampling effort. All GIS (packages “Raster,” “rgdal,” and “sp”) and statistical operations (packages “Hmisc,” “fossil,” “vegan,” and “spdep”) were performed in R (R Core Team 2013).

### Correcting sampling bias

The simplest method for estimating diversity is to calculate species richness (Peet 1974). However, species richness is highly influenced by sample effort and size (Hellmann and Fowler 1999). Many different methods for correcting sampling bias have been developed to estimate species richness. Here we use seven different methods of varying complexity to examine the extent to which application of these methods results in improved species richness estimates.

The Margalef richness index (Margalef 1958) is a simple method for correcting sampling bias derived from the semi-log relationship first proposed by Fisher et al. (1943) and following the formula:

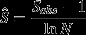
where *Ŝ* is the estimated species richness, *S*_obs_ is the number of species in a sample, and *N* is the total number of specimens in a sample, here defined as a grid cell. The Margalef index standardizes the number of species in a sample in relation to the number of observations. However, the Margalef index has also been known to be sensitive to the number of samples despite being meant to correct for sampling bias (Gamito 2010).

Chao (Chao 1984) developed a nonparametric estimate based on the following equation:

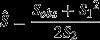
where *S*_obs_ is the observed species richness and *S*_1_ and *S*_2_ are the number of species with only one and two specimens, respectively. Chao has been shown to seriously underestimate the number of species for areas of high species richness with low sampling (Ugland and Gray 2004).

Among the more computer-intensive methods are nonparametric resampling procedures (Quinn and Keough 2002). One of these, the second-order Jackknife procedure, estimates the species richness as a function of the number of rare species in a sample by subsetting the data without replacement to species with only one or two specimens following the formula:

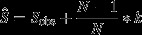
where *S*_obs_ is the observed species richness, *N* is the number of specimens within a sample, and *k* is the number of rare species in the sample defined as the subset of species with only one or two specimens (Heltshe and Forrester 1983).

Efron (1981) first proposed the bootstrap estimator where subsamples of size *N* are randomly selected from *N* specimens with replacement (Hellmann and Fowler 1999) following the formula:

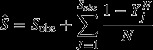
where *S*_obs_ is the total number of species, *Y*_j_ is the number of specimens of species *j*, and *N* is the number of specimens within a sample (Smith and Van Belle 1984).

Hill numbers can be used to estimate standardized species richness with integrated curves that link rarefaction and prediction on the basis of sampling completeness (Chao et al. 2014) following the formula:

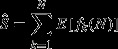
where *E*[*f*_*k*_(*N*)] is the expected number of species represented by exactly *k* specimens in a sample of *N* specimens. Rarefaction curves tend to converge at low sample sizes, which can result in imprecise richness estimates, and consequently, samples with a low number of specimens should be excluded (Gotelli and Colwell 2005). However, setting the criterion of specimen size too high will exclude many samples and the size of the subsample should be a reasonable compromise (Jiménez et al. 2009). We chose to exclude cells with <100 specimens.

With rarefaction, a subsample of size *n* is drawn randomly from the original sample and the expected species richness is calculated as:

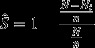
where *N* is the number of specimens within a sample, *n* is the number of specimens in the subsample, and *N*_i_ the specimens of the ith species (Hurlbert 1971). For our rarefaction procedure, we estimated species richness for subsets of data by excluding cells with fewer than 100, 500, and 1000 samples and reran the regression models for each of these subsets at all three spatial scales to evaluate the influence of sampling effort.

As a measure of sampling completeness, we constructed smoothed species accumulation curves from rarefaction with random subsampling for cells with at least 100 specimens at the 50-km scale. Following Yang et al. (2013), we used the slope of the last 10% of the curve as a proxy of sampling completeness. A shallow slope indicates saturation of species richness with sampling, and we define grid cells with slope values ≤0.05 as well sampled and those with slope values >0.05 as under-sampled.

### Statistics

We analyzed the relation between the environmental drivers and species richness with a set of multiple least squares regression (OLS) and spatial autoregressive (SAR) models. All variables were standardized before running the analysis to allow direct comparison of parameter estimates. Model performance was evaluated with the *R*^2^ value for OLS models and Nagelkerke’s pseudo *R*^2^ value for SAR models (Nagelkerke 1991). Regression analyses were repeated for each spatial resolution, 10 × 10, 25 × 25, and 50 × 50 km, to quantify the scale dependency of parameter coefficients. SAR models were included to account for spatial autocorrelation. Spatial autocorrelation could be present in the response variable (species richness) where the grids are considered independent sampling units (Colwell et al. 2012), when in fact cells in close proximity are likely to be more alike than what is expected at random.

To further evaluate the relationship between species richness and sampling effort, we calculated the pairwise Pearson’s product-moment correlations between all the different measures of species richness and the number of specimens at all the different grid sizes.

## Results

### Spatial patterns of species richness

**Figure 2:**
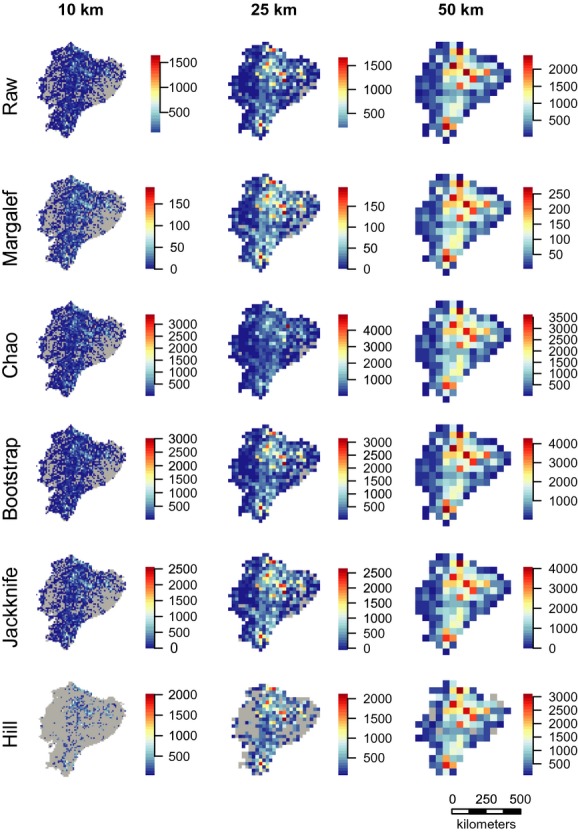
Six measures of species richness at 10-, 25-, and 50-km grid cells. Species richness was calculated as the raw number of species within a grid cell, estimated with the Margalef diversity index, the Chao estimator, the bootstrapping and jackknife resampling methods, and combined rarefaction and extrapolation with Hill numbers (see Materials and Methods for details). Gray indicates cells lacking observations. Projection: Lambert azimuthal equal-area.

**Figure 3:**
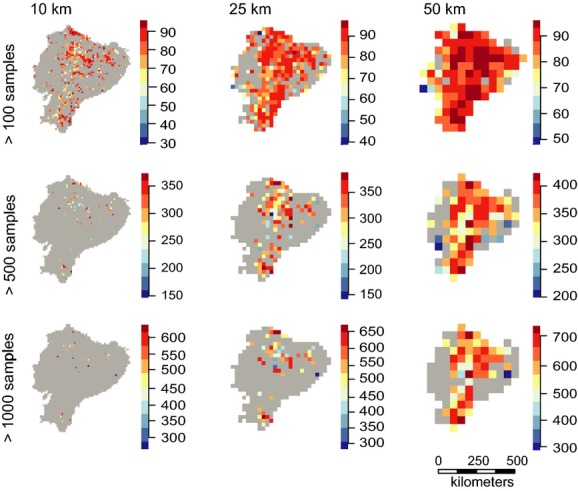
Rarefied species richness at 10-, 25-, and 50-km grid cells. Species richness was calculated as the raw number of species within a grid cell, estimated with the criterion of >100, >500, and >1000 observations per cell at each scale (see Materials and Methods for details). Gray shows cells lacking observations. Species observations were projected to the Lambert Azimuthal equal-area projection before being rasterized to avoid any effect of area on species richness estimates.

Spatial coverage, as indicated by the number of specimens within a cell, improved with increasing grid cell size (Fig. 1). The least sampled areas were the western coastal region and the eastern Amazonian region. Species richness showed a distinct spatial pattern across Ecuador, peaking in the central Andean region and decreasing in the western coastal and eastern Amazonian regions (Fig. 2). The same spatial pattern was evident at all resolutions. The spatial pattern of raw and estimated species richness mirrored the spatial patterns of number of specimens (Figs.2), with the exception of rarefied species richness (Fig. 3).

### Species richness and environmental drivers

The models of species richness containing topographic heterogeneity consistently performed better or as well as the models based on temperature, and in the following, we only present results derived from the models based on topographic heterogeneity (see Table S2 for results from the temperature models). Increased spatial coverage at higher grid size was reflected in improved model fit although the variable coefficients and *R*^2^ values in some cases changed only slightly. Excluding under-sampled cells did not affect the interpretation of the regressions, so we only show results from the full dataset (see Table S1 for results from the regressions with subsets of the data). Both *R*^2^s for the OLS and pseudo *R*^2^s for the SAR models increased (6–43%) with increasing grid size (Table 1). The number of specimens per grid cell was consistently the most important predictor variable of species richness for the methods of least complexity. Topographic heterogeneity and annual precipitation had similar low-to-moderate correlations with species richness for these methods. The results of the rarefaction procedure differed from the other methods by having lower *R*^2^s, but also by having much higher relative coefficient values for the environmental predictors and lower coefficient values for sampling (Table 1). The relationships were also scale dependent, and the effect of topographic heterogeneity and annual precipitation increased with grain size with a more than 50% increase going from 10 to 50 km. The effect of sampling decreased with increasing scale, opposite to what we found for the environmental predictors.

**Table 1:** Standardized parameter estimates from OLS and SAR models (model set 1)

	Topography		Precipitation		Sampling		*r* ^2^	
	OLS	SAR	OLS	SAR	OLS	SAR	OLS	SAR
Raw								
10	0.07***	0.07***	0.07***	0.08***	0.92***	0.90***	0.88	0.88
25	0.13***	0.13***	0.09***	0.09***	0.92***	0.91***	0.94	0.94
50	0.24***	0.24***	0.16***	0.16***	0.85***	0.84***	0.94	0.94
Margalef								
10	0.11***	0.10***	0.11***	0.11***	0.86***	0.84***	0.79	0.80
25	0.18***	0.18***	0.13***	0.13***	0.88***	0.86***	0.89	0.90
50	0.30***	0.31***	0.20***	0.21***	0.79***	0.78***	0.91	0.91
Chao								
10	0.15***	0.14***	0.20***	0.20***	0.61***	0.60***	0.45	0.45
25	0.30***	0.22***	0.26***	0.27***	0.66***	0.65***	0.61	0.61
50	0.35***	0.36***	0.27***	0.29***	0.74***	0.73***	0.88	0.88
Bootstrap								
10	0.08***	0.07***	0.07***	0.08***	0.92***	0.90***	0.87	0.87
25	0.14***	0.14***	0.01***	0.10***	0.92***	0.90***	0.93	0.93
50	0.25***	0.25***	0.16***	0.17***	0.84***	0.83***	0.93	0.93
Jackknife								
10	0.11***	0.11***	0.13***	0.15***	0.84***	0.82***	0.75	0.76
25	0.19***	0.19***	0.16***	0.17***	0.86***	0.84***	0.87	0.87
50	0.33***	0.34***	0.24***	0.25***	0.76***	0.75***	0.89	0.89
Hill								
10	0.12***	0.15***	0.20***	0.23***	0.85***	0.83***	0.77	0.78
25	0.16***	0.16***	0.17***	0.17***	0.90***	0.89***	0.89	0.89
50	0.29***	0.29***	0.22***	0.23***	0.80***	0.80***	0.91	0.91
*Rarefied*								
>100								
10	0.21***	0.28***	0.41***	0.46***	0.25***	0.23***	0.20	0.22
25	0.41***	0.41***	0.50***	0.50***	0.28***	0.28***	0.34	0.34
50	0.55***	0.56***	0.56***	0.55***	0.25**	0.25**	0.52	0.52
>500								
10	0.08	0.19	0.20	0.33*	0.31**	0.31**	0.13	0.16
25	0.39***	0.39***	0.54***	0.54***	0.39***	0.38***	0.40	0.40
50	0.63***	0.63***	0.57***	0.61***	0.32***	0.34***	0.56	0.56
>1000								
10	0.32	0.31	0.35	0.32	0.31	0.31	0.20	0.20
25	0.42*	0.43**	0.44*	0.46*	0.39**	0.38***	0.31	0.32
50	0.71***	0.71***	0.67***	0.67***	0.37**	0.36***	0.43	0.43

Standardized parameter coefficients from OLS and SAR regressions for seven measures of species richness, each modeled at three different resolutions (10, 25, and 50 km). Topography refers to topographic heterogeneity, precipitation is annual precipitation, and sampling is the number of herbarium specimens. Also shown are the *r*-squared values from the OLS models and the Nagelkerke pseudo *r*-squared values from the SAR models. For sample sizes (number of cells), see Table 2. ^*^P < 0.05, ^*^^*^P < 0.01, ^*^^*^^*^ *P* < 0.001.

### Performance of richness estimators

**Figure 4:**
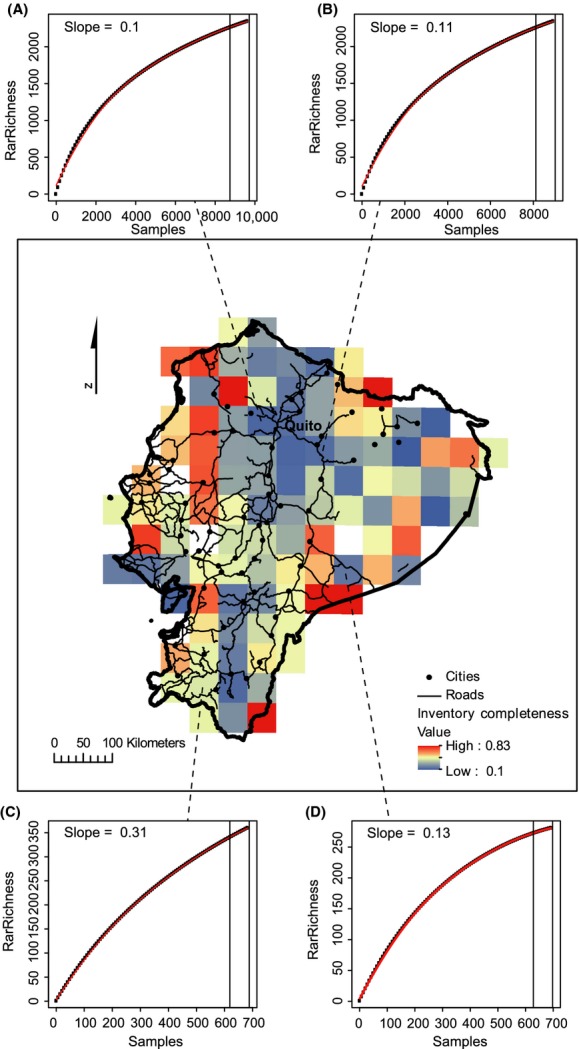
Inventory completeness across Ecuador. Inventory completeness was calculated as the slope of the last 10% of species accumulation curves for grid cells with at least 100 samples at the 50-km grid scale. A slope >0.05 indicates insufficient sampling which is evident for all cells. (A–D) show species accumulation curves for four select cells with (A) and (B) being the cells with the highest number of samples and (C) and (D) being the cells with the number of samples closet to the median. Projection: Lambert azimuthal equal-area.

The correlation between the number of specimens and the number of species was very high (Table 2) and equal or only slightly lower for the sampling-bias-corrected richness estimators compared to the raw species richness. The richness estimates of different methods were also highly correlated (Table S3). The correlation between the number of specimens and rarefied richness estimates was noticeably lower than any other measure of species richness (average Pearson correlation 0.40 for rarefied richness and 0.93 for all other measures, Table 2). The number of species increased almost linearly with number of specimens and only slightly approximated an asymptotic decline at the 50-km cell size (Fig. S1). Most species had only been sampled a few times (95% <50 specimens, Fig. S2), and half the species had <5 specimens (Fig. S2). Severe spatial under-sampling was evident with even the best sampled cells having a slope of >0.05 in the last 10% of the rarefied species accumulation curves (Fig. 4). The richness estimators performed similarly to the raw species richness in terms of both model fit and *P* values of parameter estimates for the richness–environment relations with the exception of rarefied species richness. The rarefied richness estimates had poorer model fit, but a noticeably lower correlation with sampling compared to the other sampling-bias-correction methods as well as a stronger correlation with the environmental predictors (Table 1).

**Table 2:** Pearson correlation between richness estimates, raw richness, and observations

	Observations			Richness		
	10	25	50	10	25	50
Raw	0.93	0.96	0.95	1	1	1
Margalef	0.88	0.93	0.92	0.99	0.61	0.44
Chao	0.90	0.94	0.95	0.80	0.38	0.24
Bootstrap	0.93	0.96	0.95	1.00	0.59	0.42
Jackknife	0.93	0.96	0.95	1.00	0.59	0.41
Cells	1845	425	125	1845	425	125
Hill	0.86	0.93	0.93	0.98	0.99	1.00
Cells	465	259	103	465	259	103
Rarefied						
>100	0.28	0.38	0.47	0.50	0.52	0.61
Cells	465	259	103	465	259	103
>500	0.33	0.46	0.51	0.69	0.69	0.71
Cells	82	103	73	82	103	73
>1000	0.32	0.44	0.42	0.75	0.78	0.68
Cells	27	53	54	27	53	54

Correlations between seven measures of species richness and number of observations and raw species richness for three different grid sizes. Cells show the sample size (here the number of grids cells).

## Discussion

Identification of the underlying drivers behind geographic patterns of species richness has long been a key research focus in ecology and biogeography (Hawkins et al. 2003) but requires accurate species richness estimates. Here we estimated species richness for a tropical biodiversity hotspot at three different spatial resolutions using seven different sampling-bias-correction methods. Species richness across Ecuador showed a clear geographic gradient peaking along the central Andean region (Fig. 2). However, sampling effort was consistently the most important predictor of species richness at all scales, except for rarefaction, indicating that the other methods are not able to overcome the influence of variation in sampling intensity (Table 1). This suggests that any geographic patterns observed are strongly influenced by sampling and should be interpreted with caution including correlations with environmental predictors. Our results show that for data of this kind rarefaction is the most reliable method for species richness estimation.

### Geographic patterns of species richness

The peak in species richness along the Andean region (Figs. 1, 2) could be explained by the high topographic heterogeneity in the area consistent with the hypothesis that high topographic complexity promotes habitat diversity and higher species richness (Distler et al. 2009). Cells in the Andean region of Ecuador cover a highly complex topographic area and are more likely to contain different habitat types, which may in turn result in higher species richness numbers, when compared to the more flat Coastal and Amazonian regions. The importance of topographic heterogeneity as a driver of species richness has already been confirmed by results from other authors (Gentry 1988; Kreft and Jetz 2007; Svenning et al. 2010). However, the Andes is recognized as a biodiversity hotspot, mainly due to the high number of small-range endemic species (Myers et al. 2000). Rahbek (1995) showed in a meta-study, consisting of mainly unstandardized data, that the elevation-richness gradient peaks at mid-altitude. The decline with increasing elevation has been attributed to increasingly unfavorable climatic conditions. The same pattern had previously been found for a small subsample of Ecuadorian plant species monographs with high species richness at mid-altitude on both the eastern and western sides of the Andes (Balslev 1988) and confirmed by a country-wide inventory of all vascular plants (Jørgensen and León-Yánez 1999).

Sparseness of specimens appears to obscure many underlying patterns. The western coastal region of Ecuador has very low numbers of specimens and species richness. Early deforestation in especially the central and southern parts of this area is likely to have depleted the natural vegetation and caused this pattern (Dodson and Gentry 1991). The lowland tropical rainforest of the Amazonian region has often been mentioned as an area of extremely high species richness (Schulman et al. 2007). In fact, the eastern tropical rainforests of Ecuador have been shown to be the most species-rich part of the country (Bass et al. 2010) with tree species richness alone reaching >1100 for a fully censused 0.5 × 0.5 km plot (Valencia et al. 2004). However, this pattern does not appear on our maps of species richness, even though a large part of Eastern Ecuador is lowland tropical rainforest. This area is also characterized by being highly unaccessible, and it is highly likely that the low species richness indicated on our maps is a consequence of insufficient sampling. In contrast, most of the best sampled areas are in close proximity of the capital, Quito, which further emphasizes the effect of accessibility on sampling effort (Fig. 4) and supports results from other studies (Reddy and Da 2003; Loiselle et al. 2007). This result is also not surprising considering our cells showed no sign of having reached the asymptote on the species accumulation curve (Fig. 4). This issue is particularly visible in the highest resolution maps at the 10-km scale, which shows large areas of both regions without any specimens at all (Fig. 2), particularly for rarefied richness (Fig. 3). We also see a concentration of specimens around the other two cities with major herbariums, Loja and Guayaquil (Fig. 1), indicating an effect of higher sampling by experienced botanists (Bebber et al. 2012). Despite strong evidence for sampling bias affecting the patterns of species richness here, we also consider at least part of the spatial pattern is caused by true differences in species richness. The scale of this study allows for comparison of gamma diversity, which is expected to be relatively high in the Andes due to high beta diversity, that is, species turnover caused by the heterogeneous environmental conditions. The Amazonian basin has comparably lower beta diversity and, therefore, also gamma diversity, caused by lower environmental heterogeneity. This pattern is confirmed by a count of all herbarium specimens from Ecuador which found that twice as many species were registered in the Andean region (Jørgensen and León-Yánez 1999). This difference is also seen in our maps, but it is nevertheless clear that the coastal and Amazonian regions are under-sampled as evidenced by the many grid cells with no or only a few samples.

### Estimator effect on drivers of species richness

We found a strongly scale-dependent relationship between environmental drivers and species richness. Topographic heterogeneity was positively correlated with species richness and consistently increased in importance with increasing grain size to be the strongest environmental predictor at the 50-km scale (Table 1). We also found a positive correlation between species richness and annual precipitation, but the strength of the relationship was slightly lower than for topographic heterogeneity. Sampling effort per grid cell was consistently a strong predictor of species richness (up to 24 times higher than the environmental predictors) across all spatial scales (Table 1) and is likely to be more important than real scale dependence of environmental drivers when using this kind of data. This can explain why we for most methods we did not see the strong effect of the environmental predictors that has been found in so many other studies and for different scales (Gentry 2001; Field et al. 2009). Underestimation of the importance of environmental predictors when sampling bias has strong effects on species richness estimates is likewise reported in a study from China (Yang et al. 2013).

### Performance of richness estimators

Our results show sampling effort to be the strongest predictor of variation in plant species richness in Ecuador with the exception of rarefied species richness estimates (Table 1). We also found a high correlation (0.86–0.96) between the number of specimens per grid cell and estimated species richness across all scales for the nonrarefaction methods (Table 2). This shows that the other methods we used are under most circumstances unable to remove the effect of sampling bias, resulting in unreliable species richness estimates. This finding is supported by our plots of species richness as a function of the number of observations (Fig. S1). The expected relationship would be an asymptotical decline of species richness as sampling reaches a level of saturating species richness (Colwell et al. 2012). Looking at the raw species richness as grain size and number of specimens increase, the relationship only slightly approximates an asymptote, indicating that even at the coarsest scale, Ecuador is greatly under-sampled despite having a very high overall collection density. The plots of the Margalef-, Bootstrap- and Jackknife-estimated species richness show an almost identical relationship to what we found for raw species richness. This confirms the inability of most of these methods to remove the sampling bias in our data and leads us to recommend rarefaction for species richness estimation based on herbarium specimens. The models with rarefied richness did have the lowest model performance of all the richness estimators. However, this is likely caused by the much lower sample size for this estimator, as relatively few cells had enough observations to be included in the analysis (Figs.3, S3 and Table 2). Interestingly, rarefaction was the only method resulting in stronger effects of environmental predictors than sampling effort on species richness (Table 1). Furthermore, the correlation between the number of specimens and estimated species richness was also lower for the rarefied richness estimates than any of the other methods (Table 2). This indicates that rarefaction is the only one of our chosen methods to effectively reduce the impact of sampling bias in this type of dataset.

Insufficient and uneven sampling remains a major impediment to understanding the patterns and determinants of species richness in the world’s biodiversity hotspots, even for a relatively well-sampled country such as Ecuador. Sampling was inadequate at all scales, and strong geographic biases in sampling intensity limited our ability to compare regions or assess the influence of environmental predictors on species richness. Spatial coverage could be greatly improved by focussing sampling efforts in the western coastal and eastern Amazonian regions of Ecuador; especially more generalized sampling is needed to improve the data as most species were greatly under-sampled and had fewer than 20 specimens (Fig. S2). In particular, establishment of a network of plots or transects with complete registration of all vascular plants could greatly improve our understanding of alpha, beta, and gamma diversity. Although this process is very costly and time-consuming, the gained knowledge would be well worth the effort as plot richness can be used to extrapolate species richness at larger scales (Plotkin et al. 2000). Even for the best sampled cell, we found evidence of severe under-sampling (Fig. 4A, slope of last 10% = 0.1). This leads us to conclude that much more sampling or different methods are needed before we can provide reliable richness estimates for Ecuador or any other country with similar or worse data situations.

**Figure 5:**
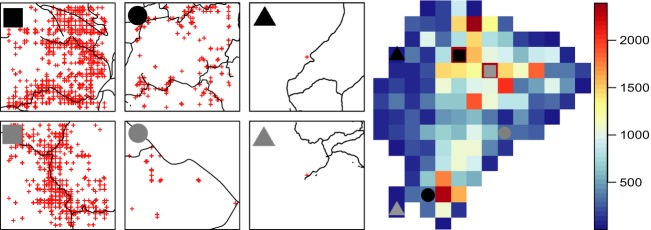
Position of georeferenced specimens in relation to roads within selected 50-km grid cells. The six chosen cells represent the two cells with the highest, closest to the median, and lowest number of samples, respectively. Also shown is a raster map of Ecuador displaying the number of species per grid as well as the position of the selected grid cells (marked by the matching circles, squares, and triangles). Projection: Lambert azimuthal equal-area.

The strong and persistent relationship between species richness and sampling intensity most likely reflects strong sampling biases, with severe under-sampling in many areas and associated strong Wallacean and perhaps Linnaean shortfalls – even when standard measures intended to correct for such biases are employed. An alternative explanation would be that the best sampled areas are also the areas with the highest species richness and vice versa. This scenario is not entirely unlikely, as especially taxonomic collection activities are often concentrated in areas known to be species rich. However, many years of research in Yasuní National Park located in the lowland tropical Amazonian rainforest of eastern Ecuador have identified this area as the most diverse region in Ecuador (Bass et al. 2010), a pattern confirmed by stacking species distribution modeling (Mateo et al. 2013), but this area was on our maps characterized by low species richness for the 10-km scale with many unsampled cells (Fig. 1). The effect of accessibility was also clear in our study with observations being clustered in close proximity to roads and major cities (Figs.5).

The challenges associated with estimating species richness from georeferenced specimens have shifted attention to alternative methods. One alternative is estimation of species richness by stacking species distribution maps (Dubuis et al. 2011). This approach has proven very successful in producing reliable species distribution maps even from a limited number of specimens (Loiselle et al. 2007), but is also not without its own issues. Data on most species, especially in the tropics, consist of only few presence records making species distribution modeling difficult or impossible (Elith et al. 2006). Although modeling many species simultaneously is currently time-consuming and computationally intensive, technological advances may soon render this issue obsolete (Geen et al. 1982). However, species distribution modeling remains dependent on the underlying environmental predictors, which have been shown to be strongly scale dependent (McGill 2010), and whose selection may be subjective. Still, species distribution modeling is a valuable supplement to species richness estimations from georeferenced specimens, and databases of species distribution maps (e.g., BIEN 2013; http://bien.nceas.ucsb.edu/bien/; Map of Life, http://www.mappinglife.org) offer a valuable alternative for the estimation of species richness patterns. However, a lack of primary occurrence data remains the biggest impediment to understanding of the world’s biodiversity, and therefore, it is vital to continue sampling in areas identified as under-sampled and to update existing data with new records (Beck et al. 2012).

## Conclusion

In conclusion, bias resulting from variation in sampling effort highly affected estimation of plant species richness across Ecuador, even when standard measures intended to correct for such bias were employed. Sampling effort overshadowed the effect of environmental predictors as the dominant richness predictor for most of the estimators used. Rarefaction was the only method to remove at least some of the effect of sampling bias. To attain reliable species richness estimates for tropical biodiversity hotspots, more primary sampling of species occurrences will be required to overcome the Wallacean and Linnaean shortfalls and enhance the usefulness of existing “big data” collections for synthetic research.

## Supporting Information

**Figure S1.** Species richness as a function of sample size for the six estimators of species richness at 10, 25 and 50 km scale.

**Figure S2.** The number of observations per species.

**Figure S3.** Mean and standard deviation of rarefied richness at all scales.

**Table S1.** Standardized parameter estimates from OLS and SAR models. **Table S2.** Standardized parameter estimates from OLS and SAR models. **Table S3.** Correlation between different measures of species richness at different grid sizes.
