# Supplementary material for: Limited sampling hampers “big data” estimation of species richness in a tropical biodiversity hotspot
Source: Ecol Evol. 2015 Jan 21;5(3):807–20. doi: 10.1002/ece3.1405 (PMC4328781; doi:10.1002/ece3.1405)
Supplement: Supplementary file 4 [file ece30005-0807-sd4.docx]

## Table S1 Standardised parameter estimates from OLS and SAR models (model set 1) for subsets of the dataset created by excluding the least sampled cells

| ***>20*** | **Topography** | | **Precipitation** | | **Sampling** | | **r^2^** | |
| --- | --- | --- | --- | --- | --- | --- | --- | --- |
|  | **OLS** | **SAR** | **OLS** | **SAR** | **OLS** | **SAR** | **OLS** | **SAR** |
| **Raw** |  |  |  |  |  |  |  |  |
| **10** | 0.06*** | 0.07*** | 0.09*** | 0.10*** | 0.92*** | 0.90*** | 0.87 | 0.88 |
| **25** | 0.11*** | 0.11*** | 0.10*** | 0.10*** | 0.93*** | 0.93*** | 0.94 | 0.94 |
| **50** | 0.23*** | 0.24*** | 0.16*** | 0.17*** | 0.85*** | 0.84*** | 0.94 | 0.94 |
| **Margalef** |  |  |  |  |  |  |  |  |
| **10** | 0.09*** | 0.10*** | 0.13*** | 0.14*** | 0.87*** | 0.85*** | 0.79 | 0.80 |
| **25** | 0.15*** | 0.16*** | 0.13*** | 0.14*** | 0.89*** | 0.8*** | 0.89 | 0.89 |
| **50** | 0.30*** | 0.30*** | 0.21*** | 0.22*** | 0.79*** | 0.9*** | 0.91 | 0.91 |
| **Chao2** |  |  |  |  |  |  |  |  |
| **10** | 0.13*** | 0.13*** | 0.29*** | 0.30*** | 0.54*** | 0.53*** | 0.39 | 0.41 |
| **25** | 0.19*** | 0.19*** | 0.29*** | 0.29*** | 0.65*** | 0.65*** | 0.58 | 0.58 |
| **50** | 0.35*** | 0.35*** | 0.28*** | 0.29*** | 0.74*** | 0.73*** | 0.88 | 0.88 |
| **Bootstrap** |  |  |  |  |  |  |  |  |
| **10** | 0.07*** | 0.07*** | 0.10*** | 0.11*** | 0.92*** | 0.90*** | 0.86 | 0.87 |
| **25** | 0.12*** | 0.12*** | 0.10*** | 0.10*** | 0.93*** | 0.92*** | 0.93 | 0.93 |
| **50** | 0.24*** | 0.25*** | 0.17*** | 0.18*** | 0.84*** | 0.83*** | 0.93 | 0.93 |
| **Jackknife** |  |  |  |  |  |  |  |  |
| **10** | 0.1*** | 0.12*** | 0.17*** | 0.19*** | 0.83*** | 0.80*** | 0.74 | 0.75 |
| **25** | 0.17*** | 0.17*** | 0.17*** | 0.17*** | 0.87*** | 0.86*** | 0.86 | 0.87 |
| **50** | 0.33*** | 0.34*** | 0.25*** | 0.26*** | 0.76*** | 0.75*** | 0.89 | 0.89 |
|  |  |  |  |  |  |  |  |  |
| ***>50*** | **Topography** | | **Precipitation** | | **Sampling** | | **r^2^** |  |
|  | **OLS** | **SAR** | **OLS** | **SAR** | **OLS** | **SAR** | **OLS** | **SAR** |
| **Raw** |  |  |  |  |  |  |  |  |
| **10** | 0.07*** | 0.08*** | 0.10*** | 0.11*** | 0.92*** | 0.91*** | 0.87 | 0.87 |
| **25** | 0.11*** | 0.11*** | 0.11*** | 0.10*** | 0.93*** | 0.93*** | 0.93 | 0.94 |
| **50** | 0.23*** | 0.24*** | 0.16*** | 0.17*** | 0.85*** | 0.84*** | 0.94 | 0.94 |
| **Margalef** |  |  |  |  |  |  |  |  |
| **10** | 0.09*** | 0.10*** | 0.14*** | 0.15*** | 0.87*** | 0.86*** | 0.79 | 0.80 |
| **25** | 0.15*** | 0.15*** | 0.14*** | 0.14*** | 0.90*** | 0.90*** | 0.90 | 0.90 |
| **50** | 0.30*** | 0.30*** | 0.21*** | 0.22*** | 0.79*** | 0.79*** | 0.91 | 0.91 |
| **Chao2** |  |  |  |  |  |  |  |  |
| **10** | 0.13*** | 0.15*** | 0.30*** | 0.31*** | 0.53*** | 0.52*** | 0.37 | 0.39 |
| **25** | 0.19*** | 0.20*** | 0.30*** | 0.31*** | 0.64*** | 0.64*** | 0.55 | 0.56 |
| **50** | 0.35*** | 0.35*** | 0.28*** | 0.29*** | 0.74*** | 0.73*** | 0.88 | 0.88 |
| **Bootstrap** |  |  |  |  |  |  |  |  |
| **10** | 0.07*** | 0.08*** | 0.11*** | 0.12*** | 0.91*** | 0.90*** | 0.86 | 0.86 |
| **25** | 0.12*** | 0.12*** | 0.11*** | 0.11*** | 0.93*** | 0.93*** | 0.93 | 0.93 |
| **50** | 0.24*** | 0.25*** | 0.17*** | 0.18*** | 0.84*** | 0.83*** | 0.93 | 0.93 |
|  |  |  |  |  |  |  |  |  |
| **Jackknife** |  |  |  |  |  |  |  |  |
| **10** | 0.12*** | 0.13*** | 0.19*** | 0.21*** | 0.82*** | 0.80*** | 0.72 | 0.73 |
| **25** | 0.18*** | 0.18*** | 0.18*** | 0.19*** | 0.7*** | 0.86*** | 0.86 | 0.86 |
| **50** | 0.33*** | 0.34*** | 0.25*** | 0.26*** | 0.76*** | 0.75*** | 0.89 | 0.89 |
|  |  |  |  |  |  |  |  |  |
| ***>100*** | **Topography** | | **Precipitation** | | **Sampling** | | **r^2^** |  |
|  | **OLS** | **SAR** | **OLS** | **SAR** | **OLS** | **SAR** | **OLS** | **SAR** |
| **Raw** |  |  |  |  |  |  |  |  |
| **10** | 0.08*** | 0.11*** | 0.12*** | 0.15*** | 0.91*** | 0.90*** | 0.86 | 0.87 |
| **25** | 0.12*** | 0.12*** | 0.11*** | 0.11*** | 0.94*** | 0.93*** | 0.93 | 0.93 |
| **50** | 0.23*** | 0.23*** | 0.17*** | 0.17*** | 0.85*** | 0.85*** | 0.94 | 0.94 |
| **Margalef** |  |  |  |  |  |  |  |  |
| **10** | 0.11*** | 0.15*** | 0.16*** | 0.20*** | 0.86*** | 0.85*** | 0.79 | 0.80 |
| **25** | 0.16*** | 0.16*** | 0.14*** | 0.15*** | 0.90*** | 0.90*** | 0.90 | 0.90 |
| **50** | 0.29*** | 0.30*** | 0.22*** | 0.23*** | 0.80** | 0.79*** | 0.91 | 0.91 |
| **Chao2** |  |  |  |  |  |  |  |  |
| **10** | 0.16*** | 0.18*** | 0.34*** | 0.36*** | 0.62*** | 0.61*** | 0.50 | 0.51 |
| **25** | 0.23*** | 0.23*** | 0.32*** | 0.32*** | 0.64*** | 0.64*** | 0.55 | 0.55 |
| **50** | 0.35*** | 0.35*** | 0.30*** | 0.30*** | 0.74*** | 0.73*** | 0.88 | 0.88 |
| **Bootstrap** |  |  |  |  |  |  |  |  |
| **10** | 0.08*** | 0.11*** | 0.13*** | 0.16*** | 0.91*** | 0.90*** | 0.85 | 0.86 |
| **25** | 0.12*** | 0.12*** | 0.11*** | 0.11*** | 0.93*** | 0.93*** | 0.93 | 0.93 |
| **50** | 0.24*** | 0.25*** | 0.18*** | 0.19*** | 0.84*** | 0.84*** | 0.93 | 0.93 |
| **Jackknife** |  |  |  |  |  |  |  |  |
| **10** | 0.1*** | 0.16*** | 0.23*** | 0.27*** | 0.79*** | 0.78*** | 0.70 | 0.71 |
| **25** | 0.1*** | 0.19*** | 0.20*** | 0.20*** | 0.87*** | 0.86*** | 0.86 | 0.86 |
| **50** | 0.33*** | 0.33*** | 0.26*** | 0.27*** | 0.76*** | 0.76*** | 0.89 | 0.89 |

## Table S2 Standardised parameter estimates from OLS and SAR models (model set 2)

|  | **Elevation** | | **Precipitation** | | **Sampling** | | **r^2^** | |
| --- | --- | --- | --- | --- | --- | --- | --- | --- |
|  | **OLS** | **SAR** | **OLS** | **SAR** | **OLS** | **SAR** | **OLS** | **SAR** |
| **Raw** |  |  |  |  |  |  |  |  |
| **10** | -0.06*** | -0.06*** | 0.07*** | 0.07*** | 0.92*** | 0.92*** | 0.88 | 0.88 |
| **25** | 0.07*** | 0.08*** | 0.04** | 0.05* | 0.94*** | 0.91*** | 0.93 | 0.93 |
| **50** | 0.20*** | 0.20*** | 0.13*** | 0.13*** | 0.88*** | 0.87*** | 0.93 | 0.93 |
| **Margalef** | |  |  |  |  |  |  |  |
| **10** | 0.11*** | 0.10*** | 0.12*** | 0.13*** | 0.86*** | 0.84*** | 0.79 | 0.80 |
| **25** | 0.11*** | 0.12*** | 0.05** | 0.06* | 0.90*** | 0.86*** | 0.88 | 0.89 |
| **50** | 0.26*** | 0.27*** | 0.17*** | 0.17*** | 0.83*** | 0.82*** | 0.90 | 0.90 |
| **Chao2** |  |  |  |  |  |  |  |  |
| **10** | 0.10*** | 0.08* | 0.19*** | 0.21*** | 0.61*** | 0.60*** | 0.43 | 0.45 |
| **25** | 0.15*** | 0.16*** | 0.17*** | 0.18*** | 0.68*** | 0.67*** | 0.59 | 0.60 |
| **50** | 0.28*** | 0.28*** | 0.22*** | 0.23*** | 0.79*** | 0.77*** | 0.86 | 0.86 |
| **Bootstrap** | |  |  |  |  |  |  |  |
| **10** | 0.07*** | 0.06*** | 0.08*** | 0.10*** | 0.91*** | 0.90*** | 0.87 | 0.87 |
| **25** | 0.08*** | 0.08*** | 0.04** | 0.05* | 0.93*** | 0.91*** | 0.92 | 0.93 |
| **50** | 0.21*** | 0.22*** | 0.14*** | 0.14*** | 0.87*** | 0.86*** | 0.93 | 0.93 |
| **Jackknife** | |  |  |  |  |  |  |  |
| **10** | 0.08*** | 0.06* | 0.13*** | 0.15*** | 0.84*** | 0.82*** | 0.75 | 0.76 |
| **25** | 0.11*** | 0.12*** | 0.08*** | 0.10** | 0.88*** | 0.84*** | 0.86 | 0.86 |
| **50** | 0.27*** | 0.28*** | 0.19*** | 0.20*** | 0.81*** | 0.79*** | 0.88 | 0.88 |
| **Hill** |  |  |  |  |  |  |  |  |
| **10** | 0.04 | 0.07 | 0.11** | 0.13** | 0.85*** | 0.84*** | 0.76 | 0.77 |
| **25** | -0.05 | -0.04 | 0.12*** | 0.12** | 0.92*** | 0.90*** | 0.88 | 0.88 |
| **50** | -0.17*** | -0.16** | 0.17*** | 0.19** | 0.84*** | 0.82*** | 0.88 | 0.88 |
| ***Rarefied*** |  | |  | |  | |  | |
| **>100** |  |  |  |  |  |  |  |  |
| **10** | -0.06 | -0.04 | 0.26*** | 0.30*** | 0.26*** | 0.25*** | 0.17 | 0.18 |
| **25** | 0.18** | 0.19** | 0.30*** | 0.31*** | 0.32*** | 0.31*** | 0.26 | 0.28 |
| **50** | 0.54*** | 0.54*** | 0.52*** | 0.52*** | 0.30*** | 0.29*** | 0.54 | 0.55 |
| **>500** |  |  |  |  |  |  |  |  |
| **10** | -0.65*** | -0.68*** | -0.39* | -0.47** | 0.24* | 0.25* | 0.24 | 0.25 |
| **25** | 0.12 | 0.12 | 0.32*** | 0.33*** | 0.39*** | 0.40*** | 0.31 | 0.31 |
| **50** | 0.62*** | 0.61*** | 0.56*** | 0.57*** | 0.40*** | 0.39*** | 0.56 | 0.56 |
| **>1000** |  |  |  |  |  |  |  |  |
| **10** | -0.38 | -0.26 | -0.13 | -0.32 | 0.26 | 0.31 | 0.18 | 0.19 |
| **25** | 0.09 | 0.08 | 0.17 | 0.16 | 0.42** | 0.42** | 0.23 | 0.23 |
| **50** | 0.72*** | 0.75*** | 0.69*** | 0.70*** | 0.48*** | 0.48*** | 0.44 | 0.45 |

## Table S3 Correlation between methods for species richness estimation

|  | **Raw.10** | **Mar.10** | **Chao.10** | **Boot.10** | **Jack.10** | **Hill.10** | **Rar.10.100** | **Rar.10.500** | **Rar.10.1000** | **Raw.25** | **Mar.25** | **Chao.25** | **Boot.25** | **Jack.25** | **Hill.25** | **Rar.25.100** | **Rar.25.500** | **Rar.25.1000** | **Raw.50** | **Mar.50** | **Chao.50** | **Boot.50** | **Jack.50** | **Hill.50** | **Rar.50.100** | **Rar.50.500** | **Rar.50.1000** |
| --- | --- | --- | --- | --- | --- | --- | --- | --- | --- | --- | --- | --- | --- | --- | --- | --- | --- | --- | --- | --- | --- | --- | --- | --- | --- | --- | --- |
| **Raw.10** | 1 | **0.99** | **0.76** | **1.00** | **0.98** | **0.98** | 0.50 | 0.69 | 0.75 | 0.60 | 0.59 | 0.47 | 0.60 | 0.59 | 0.55 | 0.25 | 0.28 | 0.26 | 0.42 | 0.42 | 0.41 | 0.42 | 0.41 | 0.40 | 0.23 | 0.21 | 0.18 |
| **Mar.10** | 0.99 | 1 | 0.78 | 0.99 | 0.99 | 0.99 | 0.58 | 0.77 | 0.83 | 0.61 | 0.61 | 0.52 | 0.61 | 0.61 | 0.56 | 0.29 | 0.31 | 0.29 | 0.45 | 0.44 | 0.43 | 0.45 | 0.44 | 0.42 | 0.26 | 0.23 | 0.19 |
| **Chao.10** | 0.76 | 0.78 | 1 | 0.76 | 0.82 | 0.90 | 0.73 | 0.81 | 0.85 | 0.52 | 0.52 | 0.52 | 0.52 | 0.54 | 0.52 | 0.32 | 0.39 | 0.39 | 0.38 | 0.38 | 0.40 | 0.38 | 0.39 | 0.36 | 0.27 | 0.26 | 0.23 |
| **Boot.10** | 1.00 | 0.99 | 0.76 | 1 | 0.98 | 0.99 | 0.51 | 0.70 | 0.76 | 0.60 | 0.59 | 0.47 | 0.60 | 0.59 | 0.55 | 0.26 | 0.28 | 0.26 | 0.43 | 0.42 | 0.41 | 0.42 | 0.41 | 0.40 | 0.23 | 0.21 | 0.18 |
| **Jack.10** | 0.98 | 0.99 | 0.82 | 0.98 | 1 | 0.99 | 0.63 | 0.81 | 0.85 | 0.61 | 0.60 | 0.53 | 0.60 | 0.61 | 0.56 | 0.30 | 0.34 | 0.33 | 0.43 | 0.43 | 0.43 | 0.43 | 0.43 | 0.41 | 0.26 | 0.24 | 0.21 |
| **Hill.10** | 0.98 | 0.99 | 0.90 | 0.99 | 0.99 | 1 | 0.59 | 0.77 | 0.82 | 0.54 | 0.54 | 0.52 | 0.54 | 0.55 | 0.55 | 0.38 | 0.42 | 0.32 | 0.37 | 0.37 | 0.38 | 0.37 | 0.37 | 0.37 | 0.29 | 0.29 | 0.27 |
| **Rar.10.100** | 0.50 | 0.58 | 0.73 | 0.51 | 0.63 | 0.59 | 1 | 0.95 | 0.94 | 0.36 | 0.38 | 0.43 | 0.36 | 0.41 | 0.40 | 0.60 | 0.63 | 0.49 | 0.27 | 0.28 | 0.32 | 0.27 | 0.30 | 0.29 | 0.41 | 0.38 | 0.39 |
| **Rar.10.500** | 0.69 | 0.77 | 0.81 | 0.70 | 0.81 | 0.77 | 0.95 | 1 | 0.99 | 0.31 | 0.33 | 0.39 | 0.31 | 0.36 | 0.32 | 0.55 | 0.61 | 0.59 | 0.23 | 0.26 | 0.28 | 0.24 | 0.27 | 0.25 | 0.47 | 0.47 | 0.44 |
| **Rar.10.1000** | 0.75 | 0.83 | 0.85 | 0.76 | 0.85 | 0.82 | 0.94 | 0.99 | 1 | 0.58 | 0.61 | 0.65 | 0.59 | 0.63 | 0.61 | 0.81 | 0.89 | 0.85 | 0.62 | 0.66 | 0.68 | 0.63 | 0.67 | 0.65 | 0.79 | 0.80 | 0.79 |
| **Raw.25** | 0.60 | 0.61 | 0.52 | 0.60 | 0.61 | 0.54 | 0.36 | 0.31 | 0.58 | 1 | **0.99** | **0.78** | **1.00** | **0.99** | **0.99** | 0.51 | 0.69 | 0.79 | 0.72 | 0.71 | 0.70 | 0.72 | 0.71 | 0.69 | 0.41 | 0.42 | 0.35 |
| **Mar.25** | 0.59 | 0.61 | 0.52 | 0.59 | 0.60 | 0.54 | 0.38 | 0.33 | 0.61 | 0.99 | 1 | 0.79 | 1.00 | 0.99 | 1.00 | 0.56 | 0.74 | 0.84 | 0.73 | 0.73 | 0.71 | 0.73 | 0.72 | 0.70 | 0.44 | 0.44 | 0.37 |
| **Chao.25** | 0.47 | 0.52 | 0.52 | 0.47 | 0.53 | 0.52 | 0.43 | 0.39 | 0.65 | 0.78 | 0.79 | 1 | 0.78 | 0.81 | 0.97 | 0.59 | 0.79 | 0.87 | 0.61 | 0.62 | 0.63 | 0.61 | 0.63 | 0.59 | 0.44 | 0.45 | 0.39 |
| **Boot.25** | 0.60 | 0.61 | 0.52 | 0.60 | 0.60 | 0.54 | 0.36 | 0.31 | 0.59 | 1.00 | 1.00 | 0.78 | 1 | 0.99 | 0.99 | 0.52 | 0.69 | 0.79 | 0.72 | 0.72 | 0.70 | 0.72 | 0.71 | 0.69 | 0.41 | 0.42 | 0.35 |
| **Jack.25** | 0.59 | 0.61 | 0.54 | 0.59 | 0.61 | 0.55 | 0.41 | 0.36 | 0.63 | 0.99 | 0.99 | 0.81 | 0.99 | 1 | 1.00 | 0.58 | 0.77 | 0.87 | 0.72 | 0.72 | 0.71 | 0.72 | 0.72 | 0.70 | 0.45 | 0.47 | 0.40 |
| **Hill.25** | 0.55 | 0.56 | 0.52 | 0.55 | 0.56 | 0.55 | 0.40 | 0.32 | 0.61 | 0.99 | 1.00 | 0.97 | 0.99 | 1.00 | 1 | 0.57 | 0.74 | 0.84 | 0.66 | 0.66 | 0.65 | 0.66 | 0.65 | 0.66 | 0.43 | 0.45 | 0.39 |
| **Rar.25.100** | 0.25 | 0.29 | 0.32 | 0.26 | 0.30 | 0.38 | 0.60 | 0.55 | 0.81 | 0.51 | 0.56 | 0.59 | 0.52 | 0.58 | 0.57 | 1 | 0.93 | 0.95 | 0.43 | 0.46 | 0.50 | 0.44 | 0.48 | 0.46 | 0.67 | 0.60 | 0.52 |
| **Rar.25.500** | 0.28 | 0.31 | 0.39 | 0.28 | 0.34 | 0.42 | 0.63 | 0.61 | 0.89 | 0.69 | 0.74 | 0.79 | 0.69 | 0.77 | 0.74 | 0.93 | 1 | 0.99 | 0.50 | 0.54 | 0.58 | 0.51 | 0.57 | 0.54 | 0.74 | 0.73 | 0.73 |
| **Rar.25.1000** | 0.26 | 0.29 | 0.39 | 0.26 | 0.33 | 0.32 | 0.49 | 0.59 | 0.85 | 0.79 | 0.84 | 0.87 | 0.79 | 0.87 | 0.84 | 0.95 | 0.99 | 1 | 0.58 | 0.63 | 0.65 | 0.59 | 0.64 | 0.62 | 0.81 | 0.83 | 0.83 |
| **Raw.50** | 0.42 | 0.45 | 0.38 | 0.43 | 0.43 | 0.37 | 0.27 | 0.23 | 0.62 | 0.72 | 0.73 | 0.61 | 0.72 | 0.72 | 0.66 | 0.43 | 0.50 | 0.58 | 1 | **1.00** | **0.98** | **1.00** | **0.99** | **1.00** | **0.61** | 0.71 | 0.68 |
| **Mar.50** | 0.42 | 0.44 | 0.38 | 0.42 | 0.43 | 0.37 | 0.28 | 0.26 | 0.66 | 0.71 | 0.73 | 0.62 | 0.72 | 0.72 | 0.66 | 0.46 | 0.54 | 0.63 | 1.00 | 1 | 0.98 | 1.00 | 1.00 | 1.00 | 0.65 | 0.76 | 0.73 |
| **Chao.50** | 0.41 | 0.43 | 0.40 | 0.41 | 0.43 | 0.38 | 0.32 | 0.28 | 0.68 | 0.70 | 0.71 | 0.63 | 0.70 | 0.71 | 0.65 | 0.50 | 0.58 | 0.65 | 0.98 | 0.98 | 1 | 0.98 | 0.99 | 0.99 | 0.68 | 0.80 | 0.78 |
| **Boot.50** | 0.42 | 0.45 | 0.38 | 0.42 | 0.43 | 0.37 | 0.27 | 0.24 | 0.63 | 0.72 | 0.73 | 0.61 | 0.72 | 0.72 | 0.66 | 0.44 | 0.51 | 0.59 | 1.00 | 1.00 | 0.98 | 1 | 0.99 | 1.00 | 0.62 | 0.72 | 0.68 |
| **Jack.50** | 0.41 | 0.44 | 0.39 | 0.41 | 0.43 | 0.37 | 0.30 | 0.27 | 0.67 | 0.71 | 0.72 | 0.63 | 0.71 | 0.72 | 0.65 | 0.48 | 0.57 | 0.64 | 0.99 | 1.00 | 0.99 | 0.99 | 1 | 1.00 | 0.66 | 0.79 | 0.77 |
| **Hill.50** | 0.40 | 0.42 | 0.36 | 0.40 | 0.41 | 0.37 | 0.29 | 0.25 | 0.65 | 0.69 | 0.70 | 0.59 | 0.69 | 0.70 | 0.66 | 0.46 | 0.54 | 0.62 | 1.00 | 1.00 | 0.99 | 1.00 | 1.00 | 1 | 0.64 | 0.76 | 0.73 |
| **Rar.50.100** | 0.23 | 0.26 | 0.27 | 0.23 | 0.26 | 0.29 | 0.41 | 0.47 | 0.79 | 0.41 | 0.44 | 0.44 | 0.41 | 0.45 | 0.43 | 0.67 | 0.74 | 0.81 | 0.61 | 0.65 | 0.68 | 0.62 | 0.66 | 0.64 | 1 | 0.98 | 0.95 |
| **Rar.50.500** | 0.21 | 0.23 | 0.26 | 0.21 | 0.24 | 0.29 | 0.38 | 0.47 | 0.80 | 0.42 | 0.44 | 0.45 | 0.42 | 0.47 | 0.45 | 0.60 | 0.73 | 0.83 | 0.71 | 0.76 | 0.80 | 0.72 | 0.79 | 0.76 | 0.98 | 1 | 0.99 |
| **Rar.50.1000** | 0.18 | 0.19 | 0.23 | 0.18 | 0.21 | 0.27 | 0.39 | 0.44 | 0.79 | 0.35 | 0.37 | 0.39 | 0.35 | 0.40 | 0.39 | 0.52 | 0.73 | 0.83 | 0.68 | 0.73 | 0.78 | 0.68 | 0.77 | 0.73 | 0.95 | 0.99 | 1 |
